# Long-Lasting Impact of Sugar Intake on Neurotrophins and Neurotransmitters from Adolescence to Young Adulthood in Rat Frontal Cortex

**DOI:** 10.1007/s12035-022-03115-8

**Published:** 2022-11-17

**Authors:** Maria Stefania Spagnuolo, Arianna Mazzoli, Martina Nazzaro, Antonio Dario Troise, Cristina Gatto, Claudia Tonini, Mayra Colardo, Marco Segatto, Andrea Scaloni, Valentina Pallottini, Susanna Iossa, Luisa Cigliano

**Affiliations:** 1 Institute for the Animal Production System in the Mediterranean Environment, National Research Council, P.le E.Fermi 1, 80055 Portici, Italy; 2 Department of Biology, University of Naples Federico II, Complesso Universitario Monte S. Angelo, Edificio 7, Via Cintia - I-80126, Naples, Italy; 3 Department of Science, Biomedical and Technology Science Section, University Roma Tre, Rome, Italy; 4 Department of Biosciences and Territory, University of Molise, Pesche, Italy; 5 Neuroendocrinology Metabolism and Neuropharmacology Unit, IRCSS Fondazione Santa Lucia, Rome, Italy

**Keywords:** Adolescent rat, Frontal cortex, Fructose diet, Brain-derived neurotrophic factor, Neurotransmitters, Mitochondria, Inflammation

## Abstract

The detrimental impact of fructose, a widely used sweetener in industrial foods, was previously evidenced on various brain regions. Although adolescents are among the highest consumers of sweet foods, whether brain alterations induced by the sugar intake during this age persist until young adulthood or are rescued returning to a healthy diet remains largely unexplored. To shed light on this issue, just weaned rats were fed with a fructose-rich or control diet for 3 weeks. At the end of the treatment, fructose-fed rats underwent a control diet for a further 3 weeks until young adulthood phase and compared with animals that received from the beginning the healthy control diet. We focused on the consequences induced by the sugar on the main neurotrophins and neurotransmitters in the frontal cortex, as its maturation continues until late adolescence, thus being the last brain region to achieve a full maturity. We observed that fructose intake induces inflammation and oxidative stress, alteration of mitochondrial function, and changes of brain-derived neurotrophic factor (BDNF) and neurotrophin receptors, synaptic proteins, acetylcholine, dopamine, and glutamate levels, as well as increased formation of the glycation end-products *Nε*-carboxymethyllysine (CML) and *Nε*-carboxyethyllysine (CEL). Importantly, many of these alterations (BDNF, CML, CEL, acetylcholinesterase activity, dysregulation of neurotransmitters levels) persisted after switching to the control diet, thus pointing out to the adolescence as a critical phase, in which extreme attention should be devoted to limit an excessive consumption of sweet foods that can affect brain physiology also in the long term.

**Supplementary Information:**

The online version contains supplementary material available at 10.1007/s12035-022-03115-8.

## Introduction

Fructose, a reducing monosaccharide present in fruit and honey, is the major component of the two most used sweeteners, namely, sucrose and high fructose corn syrup (HFCS). In the last decades, HFCS utilization has grown, essentially because of its longer shelf life, cheaper production, and higher sweetness which increases the palatability of sugary beverages, baked and processed foods [1, 2]. The rise in the intake of diets rich in sweeteners, mostly sweetened beverages (fruit juices, alcopops and sport drinks), in young and adolescents is particularly troubling [3–6]. Indeed, this kind of diet leads to a marked increase in daily fructose consumption that, compared to a natural consumption of 16–24 g/day with fruits and honey, can reach 80 g/day, which represents the 17–20% of the daily caloric intake [7–9].

Excessive dietary sugar intake, both in early and later life, has also been associated with altered brain metabolism and behavioral functioning [10–13]. In this context, it is noteworthy that brain maturation, particularly in frontal cortex, continues approximately until early adulthood (24 years of age) [14–16], rendering this brain area particularly susceptible to developmental disruption due to early-life nutritional or environmental insults [17]. Indeed, the consumption of high-fat or high-sugar diets during adolescence was associated with both deficits in executive functioning and a reduced volume of the frontal cortical region in humans [18–21]. Therefore, the assessment of the sugar impact on brain-related outcomes is crucial, particularly during the critical windows of growth and development, when key features of brain tissue structure and function are establishing [22]. While several studies outlined the detrimental effect of fructose on hypothalamus or hippocampus [13, 23, 24], very little information is available on the impact of this sugar on frontal cortex. In this regard, we previously reported that, in both adolescent and adult rats, a short-term fructose intake affects redox homeostasis, autophagy, and the expression of synaptic markers [25]. Furthermore, long-term fructose drinking was associated with neuroinflammation, altered insulin signaling and cognitive impairment in frontal cortex [26, 27].

Whether the alterations induced by fructose intake during adolescence (postnatal day 30–60, P30–P60) are rescued returning to a healthy diet or persist until young adulthood is a critical issue that remains unexplored. Therefore, we focused on the frontal cortex of adolescent rats fed a fructose-rich (F) or control diet (C) for 3 weeks. After this period, to highlight whether the effects induced by the short-term fructose diet persist or are rescued, fructose-fed rats were fed a control diet for a further 3 weeks (FR) until young adulthood phase and compared with animals that received the control diet for the entire period (CR). Thus, we investigated putative damages induced by the sugar intake on brain-derived neurotrophic factor (BDNF), nerve growth factor (NGF), and their specific receptors, which are crucial in modulating survival and development of the nervous system, synaptic function and plasticity, learning and memory [28, 29], as well as we evaluated the sugar impact on inflammation and oxidative stress. Moreover, a mass spectrometry-based metabolomic analysis was carried out to ascertain whether fructose intake also influences the homeostasis of amino acids, amino acid derivatives and other polar metabolites, particularly the main neurotransmitters, in the frontal cortex.

## Materials and Methods

### Materials

Bovine serum albumin fraction V (BSA; catalogue n. A6588), amino acids (catalogue n. LAA21), dopamine (catalogue n. H8502), acetylcholine (catalogue n. A6625) tyramine (catalogue n. T2879), γ-aminobutyric acid (GABA, catalogue n. 03,835), DL-lysine-4,4,5,5-d_4_ dihydrochloride (*d4*-lysine, catalogue n. 489,034), formic acid (catalogue n. 5.33002), salts, and buffers were purchased from Sigma-Aldrich (St. Louis, MO, USA). *Nε*-carboxymethyllysine (CML, catalogue n. HAA2950) and *Nε*-carboxyethyllysine (CEL, catalogue n. HAA2940) were obtained from Iris Biotech (Marktredwitz, Germany). Acetonitrile (catalogue n. 1.00029), water (catalogue n. 1.15333), and ammonium formate (catalogue n. 70,221) of mass spectrometry grade were obtained from Merck (Darmstadt, Germany).

Polyvinylidene difluoride (PVDF; catalogue n. GEH10600021) and nitrocellulose membranes (catalogue n GEH10600001) were from GE Healthcare (Milan, Italy).

Fuji Super RX films (catalogue n. 47,410–19,284), FujiFilm Man-X Developer (catalogue n. 949–966) and FujiFilm Man-X Fixer (catalogue n. 949–974) were from Laboratorio Elettronico Di Precisione (Naples, Italy).

### Experimental Design

Male Wistar rats (30 days old, P30) were purchased from Charles River; Calco, Como, Italy. Animals were caged individually in a temperature-controlled room (23 ± 1 °C) with a 12-h light/dark cycle (06.30–18.30), and divided into two groups, one fed a fructose rich diet (F group), the other one fed a control diet (C group) for 3 weeks. At the end of treatment, half of the rats from each group was euthanized, while the other half received a control diet (FR and CR groups) for further 3 weeks (P72). The composition of both control and fructose rich diet is reported in Table 1. The fructose-rich diet and the control diet were isocaloric, as differing only for qualitative content of carbohydrates (Table 1).

**Table 1:** Ingredients and nutritional composition of experimental diets

Ingredients, g/100 g	Control diet	Fructose diet
Standard Chow^a^	50.5	50.5
Sunflower Oil	1.5	1.5
Casein	9.2	9.2
Alphacel	9.8	9.8
Cornstarch	20.4	–-
Fructose	–-	20.4
Water	6.4	6.4
AIN-76 mineral mix	1.6	1.6
AIN-76 vitamin mix	0.4	0.4
Choline	0.1	0.1
Methionine	0.1	0.1
Energy content and composition		
Gross Energy Density (kJ/g)	17.2	17.2
ME content (kJ/g)^b^	11.1	11.1
Proteins (% ME)	29.0	29.0
Lipids (% ME)	10.6	10.6
Carbohydrates (% ME)	60.4	60.4
Of which:		
Fructose	–-	30.0
Starch	52.8	22.8
Sugars	7.6	7.6

^a^4RF21, Mucedola, Italy

^b^ Estimated by computation using values (kJ/g) for energy content as follows: proteins 16.736, lipids 37.656, and carbohydrates 16.736. ME = metabolizable energy

At the end of dietary treatment, the animals were then euthanized by decapitation, and frontal cortex was harvested and dissected as previously described [25]. In details, the brains were quickly harvested, moved on a metal plate placed in dry ice, and washed with cold PBS to remove surface blood. In order to dissect frontal cortex, brain was cut longitudinally, into right and left hemisphere, and the olfactory bulb was removed with a cut from the medial view of the hemisphere. Finally, frontal cortex was dissected from a slice about 2.5–4.5 mm anterior to bregma, taking into account published stereotaxic atlas resources [30, 31]. Pieces of each sample were immediately snap frozen in liquid nitrogen and stored at − 80 °C for metabolomic and protein analyses. Mitochondrial oxygen consumption was immediately assessed in little aliquots of tissue as reported below. Pieces of each sample were fixed for immunofluorescence analysis. As post-mortem metabolism is known to provoke rapid and progressive changes in the levels of many compounds [32, 33], dissection of frontal cortex from each animal was performed into 1.5 min. The time of handling before freezing was standardized to minimize the effect of post-mortem metabolism*.*

### Mitochondrial Analyses

Freshly isolated frontal cortex samples were homogenized (1:1000, w/v) in Mir05 medium containing 110 mM sucrose, 60 mM K-lactobionate, 20 mM Hepes, 20 mM taurine, 10 mM KH_2_PO_4_, 6 mM MgCl_2_, 0.5 mM EGTA, and 0.1% w/v fatty acid-free BSA, pH 7.0.

Homogenates (2 mg) were transferred into calibrated Oxygraph-2 k (O2k, Oroboros Intruments, Innsbruck, Austria) 2-mL chambers. Oxygen polarography was performed at 37 ± 0.001 °C (electronic Peltier regulation), and oxygen concentration (μM) and oxygen flux (pmol O_2_ s^−^1 mL^−1^) were real-time recorded and corrected automatically for instrumental background by DatLab software (Oroboros Intruments, Innsbruck, Austria).

After addition of the homogenates, the O_2_ flux was allowed to stabilize. A substrate, uncoupler, inhibitor titration (SUIT) protocol was applied to assess qualitative and quantitative mitochondrial changes [34]. After stabilization, leak respiration supported primarily by electron flow through complex I of the respiratory chain was evaluated by adding the substrates malate (0.5 mM), pyruvate (5 mM), and glutamate (10 mM). Electron transfer was coupled to phosphorylation by the addition of 2.5 mM ADP, assessing phosphorylating respiration with electron transfer supported by complex I. Succinate (10 mM) was added to the chamber to induce maximal phosphorylating respiration with parallel electron input from complexes I and II. Oligomycin (2.5 mM) was added to assess leak respiration when substrates and ADP were provided, but ATP synthase was inhibited. Maximum capacity of the electron transport chain was obtained by addition of the uncoupler carbonyl cyanide *p*-trifluoromethoxyphenylhydrazone (FCCP, 0.5 mM). Rotenone (0.5 μM) was added to inhibit complex I; hence, the maximal capacity supported by complex II alone was determined. Residual oxygen consumption was established by addition of the inhibitor antimycin A (2.5 mM) and the resulting value was subtracted from the fluxes in each run, to correct for non-mitochondrial respiration. All samples were run in duplicates and the mean was used for analysis.

Procedures to test mitochondrial integrity were routinely carried out at the beginning of each measurement, by evaluating the stimulating effect of 10 mM exogenous cytochrome c on mitochondrial respiration in the presence of complex I- linked substrates and ADP.

### Metabolic Parameter Assay

The amount of fructose, uric acid, and glucose in frontal cortex samples were measured by colorimetric enzymatic methods, using commercial kits according to the manufacturer’s instruction (Fructose assay kit: catalogue n. FA-20, Sigma Aldrich, St. Louis, MO, USA; Uric acid kit: catalogue n. 4059, GS Diagnostics SRL, Guidonia Montecelio, Rome, Italy; Glucose assay kit: catalogue n. 4058, GS Diagnostic).

### Protein Extraction

Aliquots of frontal cortex (about 50 mg) were homogenized in seven volumes (w/v) of cold RIPA buffer, as previously published [35]. Protein concentration was titrated by the colorimetric Bio-Rad assay, based on the Bradford method [36], using the Bio-Rad dye reagent (catalogue n. 5,000,006, Bio-Rad, Hercules, CA, USA), according to the manufacturer’s instruction. Then, protein extracts were used for titrating the markers reported below by ELISA or Western blotting.

### Analysis of Tumor Necrosis Factor Alpha (TNF-Alpha)

TNF-alpha concentration was evaluated by sandwich ELISA in frontal cortex homogenates diluted 1:20 [37] using the TNF-alpha Duo-Set kit (catalogue n. DY510, R&D, DBA Italia). Data were reported as pg of TNF-alpha per mg of total proteins.

### Western Blotting

Aliquots (30 µg) of cortex proteins were resolved by electrophoresis, under denaturing and reducing conditions [38], on 12.5% (to quantify glucose transporter-5, Glut-5; glucose transporter-4, Glut-4; glial fibrillary acidic protein, GFAP; synaptophysin; brain derived neurotrophic factor, BDNF; nerve growth factor, NGF; extracellular signal-regulated kinase, Erk1/2) or 10% (post-synaptic density protein 95, PSD-95; synaptotagmin I; peroxisome proliferator-activated receptor gamma coactivator 1-alpha, PGC-1α; nerve growth factor (NGF) receptor, p75NTR; tropomyosin receptor kinase B, TrkB; tropomyosin receptor kinase A, TrkA; thyroxine hydroxylase, TH) polyacrylamide gels. Proteins were then blotted onto PVDF or nitrocellulose membrane [39], and the following washing and blocking steps were performed according to [40].

The membranes were then incubated (overnight, at 4 °C) with primary antibody dilutions, followed by incubation (1 h, at 37 °C) with the appropriate peroxidase-conjugated secondary IgGs (see Supplementary Table 1).

For loading control, β-actin or vinculin was revealed after detection of each marker. To this aim, the membranes were stripped [41] and then treated with mouse anti-β-actin IgG or with mouse anti-vinculin IgG (overnight, at 4 °C) as described in Supplementary Table 1.

The Excellent Chemiluminescent detection Kit (Westar Antares, catalogue n. XLS142, Cyanagen s.r.l., Bologna, Italy) was used for detection. Chemidoc or digital images of X-ray films exposed to immunostained membranes were used for densitometric analysis and quantification was carried out by Un-Scan-It gel software (Silk Scientific, UT, USA).

### Haptoglobin (Hpt) Evaluation

Hpt concentration in frontal cortex samples was measured by ELISA as previously reported [40]. Samples were diluted (1: 3,000, 1:10,000, 1:30,000) with coating buffer (7 mM Na_2_CO_3_, 17 mM NaHCO_3_, 1.5 mM NaN_3_, pH 9.6), and aliquots (50 µl) were then incubated (overnight, at 4 °C) in the wells of a microtiter plate (Nunc MaxiSorp, catalogue n. 44–2404-21, Thermo Fisher Scientific). Washing and blocking were carried out as previously reported [42], then the wells were incubated (1 h, at 37 °C) with 50 µl of rabbit anti-human Haptoglobin IgG (catalogue n. H8636, Sigma-Aldrich, St. Louis, MO, USA) diluted 1:500 in T-TBS (130 mM NaCl, 20 mM Tris–HCl, 0.05% Tween, pH 7.4) containing 0.25% BSA, followed by 60 µl of goat anti-rabbit horseradish peroxidase-conjugated IgG (catalogue n. GtxRb-003-DHRPX, Immunoreagents, Raleigh, NC, USA; 1:5000 dilution in T-TBS containing 0.25% BSA; 1 h, at 37 °C). Peroxidase-catalyzed color development from o-phenylenediamine was measured at 492 nm.

### Evaluation of Nitro-Tyrosine Levels, Acetylcholinesterase (AChE) and Monoamine Oxidase (MAO) Activities

Nitro-tyrosine (N-Tyr) titration was carried out by ELISA in frontal cortex homogenates as previously described [43]. Samples were diluted (1:1,500, 1:3,000, 1:6,000) with coating buffer, and aliquots (50 µl) were then incubated in the wells of a microtiter plate (overnight, at 4 °C). After washing and blocking, the wells were incubated (1 h, 37 °C) with 50 µl of rabbit anti-N-Tyr IgG (catalogue n. CVL-PAB0188, Covalab, distributed by VinciBiochem, Vinci, Italy; 1: 1000 dilution in T-TBS containing 0.25% w/v BSA) followed by 60 µl of goat anti-rabbit horseradish peroxidase-conjugated IgG (1:9,000 dilution; 1 h, at 37 °C). Peroxidase-catalyzed color development from *o*-phenylenediamine was measured at 492 nm. Data were reported as OD per milligram of total proteins.

The acetylcholinesterase **(**AChE) activity was measured in frontal cortex samples as previously described [25]. Enzyme activity was expressed as nmol/min mg protein.

The monoamine oxidase (MAO) activity was measured spectrophotometrically following the conversion of benzylamine to benzaldehyde, as previously described [44].

### Immunofluorescence Analysis

Paraffin embedded sections of frontal cortex from all the groups were stained with the phospho-cAMP response element-binding protein (p-CREB) specific monoclonal antibody (Ser 133) (87G-3) (catalogue n. 9198, Cell Signaling Technology; 1:1,000 in dilution in PBS containing 2% w/v BSA; overnight, at 4 °C), and DAPI (catalogue n. D9542, Sigma Aldrich, Saint Louis, MO, USA). For the analysis, images were acquired with × 40 magnification and 3 random fields/section per rat were analyzed using ImageJ (National Institutes of Health, Bethesda, MD, USA). Images were captured and visualized using a Nikon Eclipse E1000 microscope.

### Liquid chromatography high resolution tandem mass spectrometry (LC–MS/MS)

Polar hydrophilic compounds were analyzed by liquid chromatography high resolution tandem mass spectrometry (LC–MS/MS) as previously reported [45], with minor modifications. Frontal cortex samples (25 ± 10 mg) were dissolved in 0.390 mL of 0.1% formic acid along with 10 µL of 10 µg/mL lysine *d-4*; suspensions were accurately homogenized by using a stainless-steel disperser (IKA T10, Staufen, Germany, 3 passes, 30 s) in an ice bath. Supernatants (0.1 mL) were further purified by directly using 0.3 mL of 0.1% formic acid in acetonitrile in a protein precipitation and phospholipids removal cartridge (Phree, 1 mL, Phenomenex, Torrance, CA); eluates were collected and dried by using a centrifugal evaporator (SpeedVac, Thermo Fisher Scientific, Bremen, Germany). Dried samples were dissolved in 0.1 mL of a mixture acetonitrile: water: formic acid (50:49.9:0.1, v/v/v) and 5 µL injected into the LC–MS/MS system consisting in a linear ion trap with Orbitrap detector (LTQ Orbitrap XL) interfaced to an Ultimate 3000 RS (Thermo Fisher Scientific, Bremen, Germany). Analytes were separated through hydrophilic interaction chromatography and analyzed in data dependent scan positive ions mode for identification and quantitation.

Chromatographic separation was achieved through a silica sulfobetaine zwitterionic modified HILIC column (100 × 2.1 mm, 1.7 µm, Syncronis HILIC, Thermo Fisher, Bremen, Germany) at 35 °C. Mobile phases consisted in 0.1% formic acid in acetonitrile:water 95:5 (v/v, solvent A) and 0.1% formic acid in water (solvent B) both with 5 mM ammonium formate. Analytes (thermostated at 4 °C) were separated through the following gradient of solvent B (minutes/%B): (0/3), (3.5/3), (15.5/75), (17.5/75) at a flow rate of 0.25 mL/min. Electrospray interface (ESI) parameters were the following: spray voltage 5.0 kV, capillary voltage 21.0 V, capillary temperature 300 °C, sheath gas flow and auxiliary gas flow were 25 and 4 arbitrary units, respectively. Profile data type were acquired in full scan FTMS mode (Fourier transformed) in the mass range 75–750 *m/z*. For data-dependent scanning mode, MS/MS normalized collision energy was set to 20, activation Q 0.25, activation time 25 ms, with a 1 *m/z* isolation window, while a reject mass list was generated by injecting blank samples consisting in a mixture of acetonitrile:water:formic acid (50:49.9:0.1, v/v/v).

For compound identification, differential analysis, principal component analysis (PCA), hierarchical clustering, and identification of metabolic pathways, raw data were loaded in Compound Discoverer (v. 3.2, Thermo Fisher Scientific). The workflow included the identification of both expected and unknown metabolites; briefly, each node performed retention time alignment, expected compound detection, biotransformation, dealkylation, and dearylation products formation. Resolution and isotope pattern matching with unknown compounds detection were used across all samples with a mass accuracy below 5 ppm. FISh (fragment ion searching) scoring was applied to all expected compounds with automatic fragment annotations based on targeted and untargeted compound chemical behavior outlined in Human Metabolome Database (https://hmdb.ca/), ChemSpider (http://www.chemspider.com), mzCloud (https://www.mzcloud.org/) and KEGG pathway database (https://www.genome.jp/kegg/compound/).

According to the background in blank samples, the procedure predicted elemental compositions for all unknown compounds, while quality control samples (QC, consisting in pooled samples spiked with amino acid standards) corrected signal intensities each 10 runs. Hierarchical clustering was obtained through filtering procedures based on technical replicates coefficient of variation (CV%), retention time, and mass accuracy, excluding analytes eluting close to the solvent front, with poor response for coefficient of separation *k’*, peak shape, and spectrum purity in both full scan and data-dependent mode. The workflow included differential analysis (*p*-values, adjusted *p*-values, ratios, fold change; Supplementary Tables 2 and 3), Euclidean distance, and complete linkage method without data normalization to enhance differences among frontal cortex of groups fed different diets. Analytes based on hierarchical clustering were scaled before clustering through a z-score transformation.

For targeted analyte quantitation, a calibration curve of the compounds listed in Supplementary Table 4 was built in the range 100–5000 ng/mL by using lysine-*d4* as internal standard. Linearity and the responses of intraday and interday assays were monitored by using Xcalibur 2.1 with a mass accuracy fixed at 5 ppm (Thermo Fisher Scientific, Bremen).

### Statistical Analysis

Data were expressed as mean values ± SD. The program GraphPad Prism 9.3.1 (GraphPad Software, San Diego, CA, USA) was used to verify normal distribution of data by Shapiro–Wilk normality test and to compare groups with one-way ANOVA followed by Bonferroni post-test. *P* < 0.05 was considered significant in the reported analyses.

## Results

### Metabolic Analyses

At the end of dietary treatment, no significant variation was evident in final body weight (C rats = 295 ± 4 g; F rats = 292 ± 5 g; CR rats = 350 ± 6 g; FR rats = 367 ± 3 g). Daily energy intake showed no significant variation in F rats compared to C rats (C rats = 358 ± 5 kJ/day; F rats = 360 ± 5 kJ/day). Similarly, comparable energy intake was found in CR and FR rats (CR rats = 340 ± 4 kJ/day; FR rats = 344 ± 5 kJ/day). In addition, fasting levels of glucose were unchanged both in F rats compared to C rats (F rats = 123 ± 5 mg/dL; C rats = 130 ± 5 mg/dL), and in FR rats compared to CR rats (FR rats = 113 ± 6 mg/dL; CR rats = 121 ± 6 mg/dL). These data evidence that the impact of fructose on brain is not due to an increase of body weight or change in daily energy intake.

### Inflammation, Oxidative Stress, and Mitochondrial Activity

**Fig. 1:**
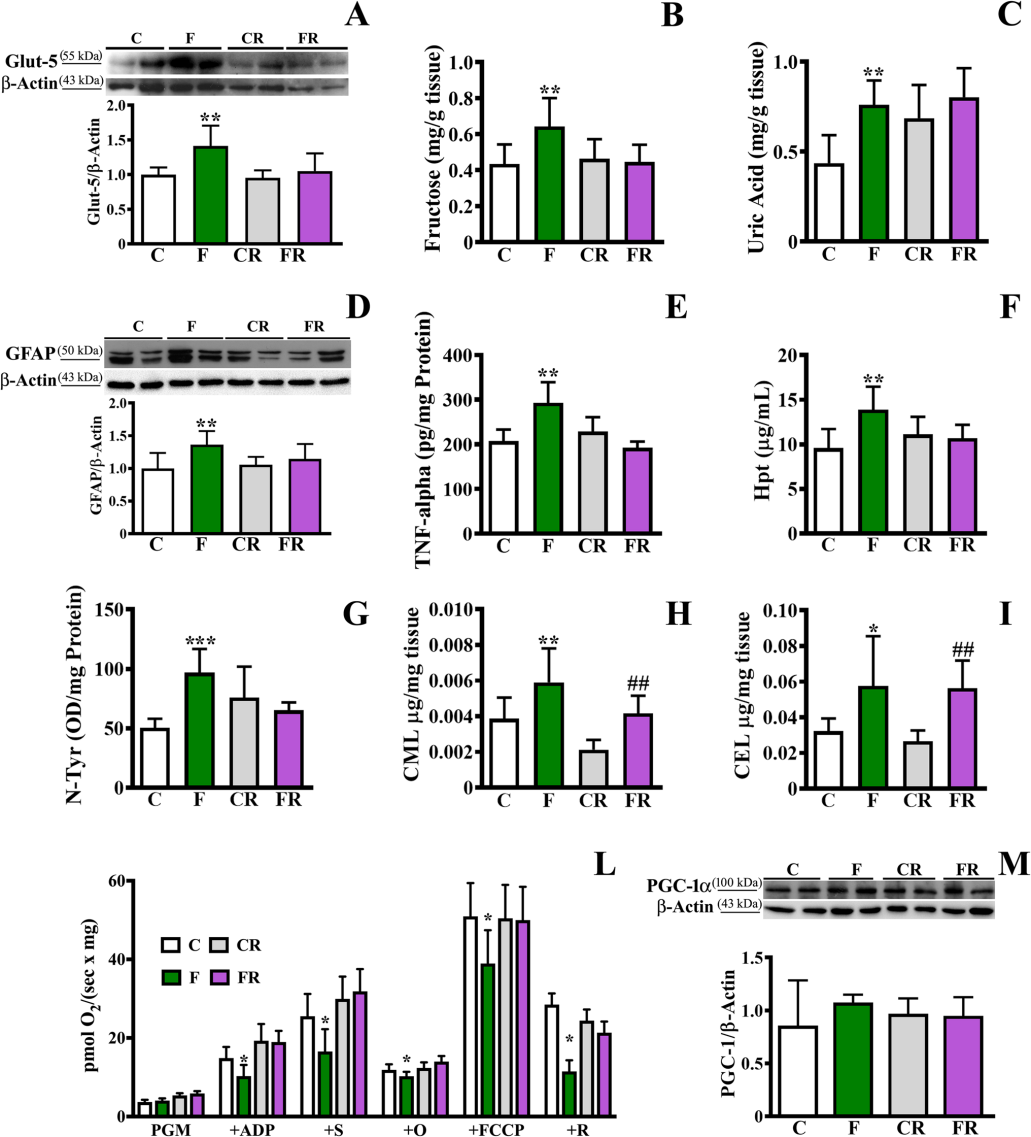
Evaluation of Glut-5, fructose, uric acid, inflammatory and oxidative stress markers, mitochondrial function in frontal cortex. (**a**) Glut-5 level (representative western blot and densitometric analysis); (**b**) fructose amount; (**c**) uric acid amount; (**d**) GFAP amount (representative western blot and densitometric analysis); (**e**) TNF-alpha concentration (titrated by sandwich ELISA); (**f**) Hpt concentration (titrated by ELISA, (**g**); N-Tyr levels (titrated by ELISA); (**h**) CML amount and (**i**) CEL amount (as determined by LC–MS/MS); (**l**) non-normalized respiration after addition of malate + pyruvate + glutamate (PMG), ADP, succinate (S), oligomycin (O), FCCP, and rotenone (R); (**m**) PGC-1α amount (representative western blot and densitometric analysis), in frontal cortex of control adolescent (C), fructose-fed adolescent (F), young-adult control rescued (CR), young-adult fructose-rescued (FR) rats. Data are the means ± SD of 8 rats/group. *P < 0.05, **P < 0.01, ***P < 0.001 versus C rats. ##*P* < 0.01 versus CR rats. Source of variation: one-way ANOVA followed by Bonferroni post-test. FCCP carbonyl cyanide *p*- trifluoromethoxyphenylhydrazone

To investigate whether the dietary treatment induces fructose transporter and metabolism, the protein expression of Glut-5, as well as the level of fructose and uric acid, one of the main products of fructose metabolism, was analyzed in frontal cortex after 3 weeks of a fructose-rich diet. The amount of Glut-5 was significantly higher [*F* (3, 28) = 7.625; *P* < 0.01] in frontal cortex of F rats compared to C rats, while this increase disappeared in FR rats (Fig. 1a). Unlike Glut-5, no significant difference was found in the level of Glut-4 between C and F rats or CR and FR (Supplementary Figure 1). According to the Glut-5 increase, a significant rise in the levels of fructose [*F* (3, 28) = 5.191; *P* < 0.01] and uric acid [*F* (3, 28) = 6.258; *P* < 0.01] was found in F rats compared to C rats, while no significant differences were found in FR rats compared to CR ones (Fig. 1b, c). The cortex level of glucose did not differ between C and F, or CR and FR rats (Supplementary Figure 1).

We then investigated the inflammatory status by measuring the protein expression of GFAP, a marker of astrogliosis, the pro-inflammatory cytokine TNF-alpha, and Hpt, a marker of inflammation very sensitive to nutritional changes [37, 40, 46]. As shown in Fig. 1d–f, an increase of inflammatory markers in frontal cortex was associated with the fructose-rich diet. As a matter of the fact, significantly higher levels of GFAP [*F* (3, 28) = 4.979; *P* < 0.01; Fig. 1d], TNF-alpha [*F* (3, 28) = 11.57; *P* < 0.01; Fig. 1e], and Hpt [*F* (3, 28) = 5.656; *P* < 0.01; Fig. 1f] were found in frontal cortex of F rats compared to C rats. Importantly, these conditions were rescued after switching to a control diet as no difference in the levels of these markers was detected between CR and FR rats.

In line with previous results obtained in the hippocampus [47], fructose feeding was also associated with an increase of N-Tyr, the footprint of protein oxidative damage induced by peroxynitrite [48] in fructose-fed rats [*F* (3, 28) = 7.283; *P* < 0.001; Fig. 1g]. The observed condition of redox imbalance was corroborated by changes in mitochondrial activity induced by fructose-rich diet. In detail, F rats showed a significant decrease in leak respiration with complex I and II-linked substrates [*F*(3, 28) = 10.36; *P* < 0.0001], in ADP-supported respiration with complex I [*F* (3,28) = 13.58; *P* < 0.0001] or complex I- and II-linked substrates [*F* (3, 28) = 11,61; *P* < 0.0001] and in FCCP-stimulated respiration with complex II [*F* (3, 28) = 52.51; *P* < 0.0001] or complex I and II-linked substrates [*F* (3, 28) = 3.70; *P* = 0.023; Fig. 1l]. The above mentioned mitochondrial dysfunction was reversed when rats were switched to a control diet. The analysis of PGC-1α was performed as a marker of mitogenesis, and no difference was observed (Fig. 1m).

We also detected higher levels of *Nε*-carboxymethyllysine (CML) [*F* (3, 28) = 10.62; *P* < 0.01; Fig. 1h] and *Nε*-carboxyethyllysine (CEL) [*F* (3, 28) = 7,756; *P* < 0.05; Fig. 1i], two advanced glycation end-products [49], both in free form, in frontal cortex of F rats compared to C rats. Notably, after switching to the control diet, the differences in N-Tyr between CR and FR rats disappeared, while CML and CEL levels persisted higher in FR compared to CR rats (*P* < 0.01).

### Neurotrophins and Synaptic Proteins

**Fig. 2:**
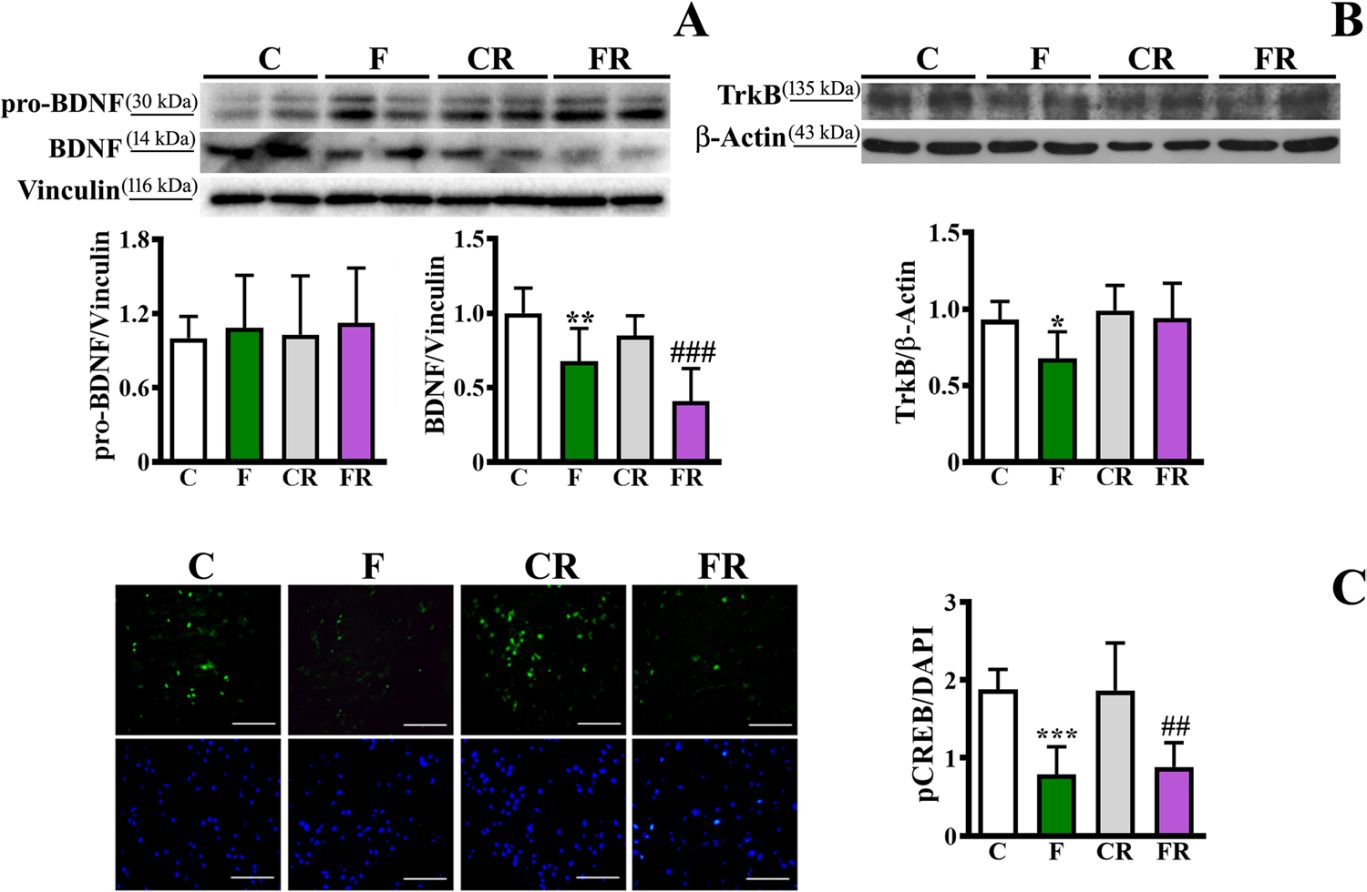
Evaluation of BDNF, TrkB and pCREB amount in frontal cortex. (**a**) pro-BDNF and BDNF levels (representative western blot and densitometric analysis), (**b**) TrkB level (representative western blot and densitometric analysis), (**c**) immunofluorescence of p-CREB (magnification 40 × , scale bar = 50 μm) in frontal cortex of control adolescent (C), fructose-fed adolescent (F), young-adult control rescued (CR), young-adult fructose-rescued (FR) rats. Data are the means ± SD of 8 rats/group. *P < 0.05, **P < 0.01 versus C rats. ##*P* < 0.01 versus CR rats. Source of variation: one-way Anova followed by Bonferroni post-test

The level of BDNF, a key cerebral factor involved in a wide range of neurophysiological processes such as neuronal survival, synaptic transmission and plasticity [50], was measured in the frontal cortex of all groups of rats. As shown in Fig. 2a, a significant fructose diet-dependent decrease of the mature form of BDNF was observed [*F* (3, 28) = 11.84; *P* < 0.01]. Notably, alterations of BDNF amount persisted after switching to a control diet, as displayed by FR rats compared to CR rats (*P* < 0.001). No difference was found in the levels of BDNF precursor (pro-BDNF), suggesting that changes in this neurotrophin are likely at post-translational level. The protein amount of TrkB, the high-affinity receptor of BDNF, was also analyzed, as its activation enhances synaptic plasticity, neuroprotection, and neurite outgrowth [50]. As shown in Fig. 2b, TrkB level was significantly lower in the frontal cortex of F rats compared to C ones [*F* (3, 28) = 3.994; *P* < 0.05], and its level in FR rats returned to values comparable to CR rats. We further investigated the extent of phosphorylation of CREB, the major downstream effector of BDNF [51, 52], as marker of BDNF signalling. In line with the decrease of BDNF, a decrease in the activating phosphorylation of CREB (p-CREB) was found in both F rats compared to C, and FR compared to CR [*F* (3, 28) = 12.42; *P* < 0.01; Fig. 2c], as assessed by immunofluorescence.

**Fig. 3:**
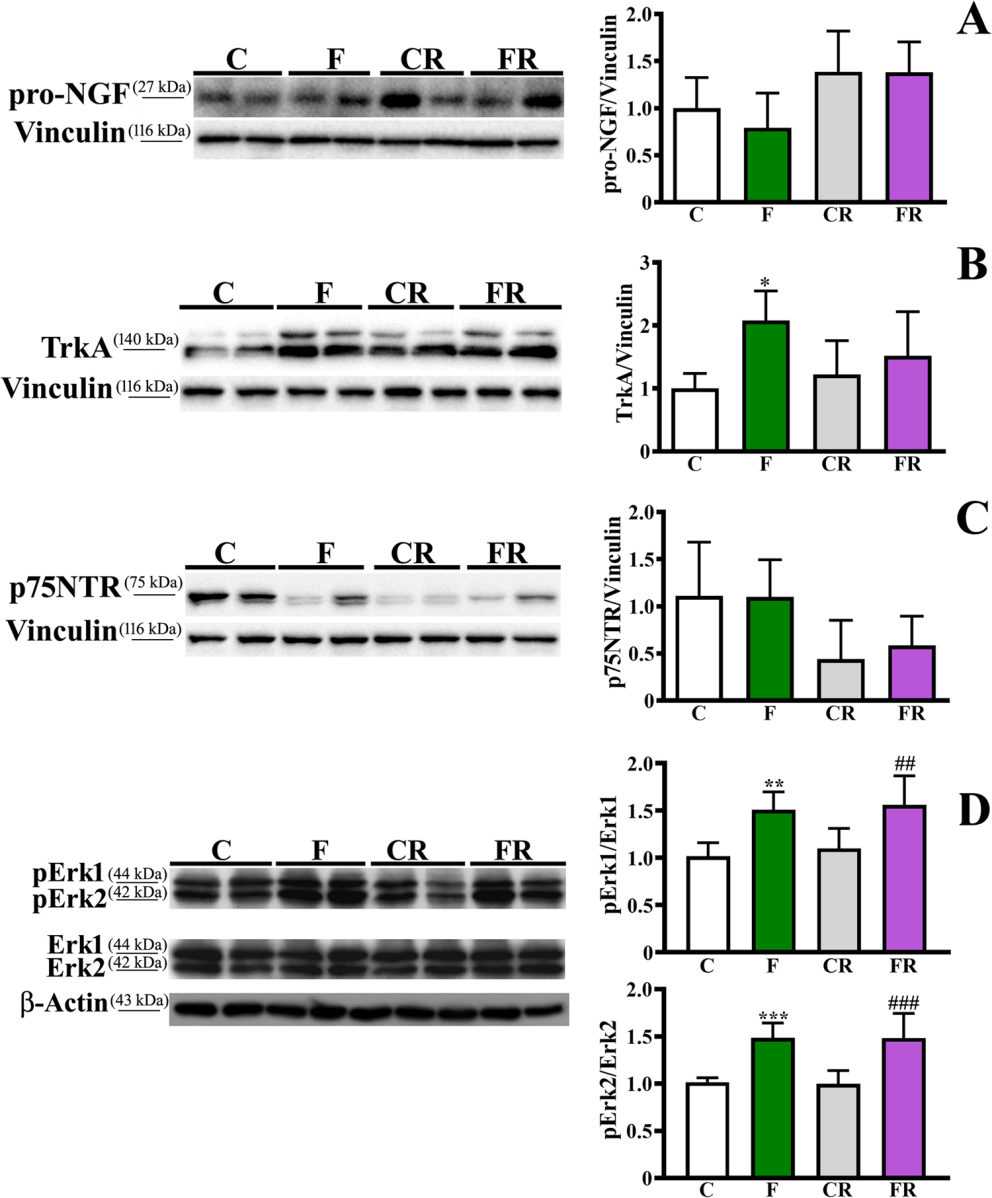
Evaluation of pro-NGF, TrkA, p75NTR, and Erk1/2 in frontal cortex. Representative western blot and densitometric analysis of (**a**) NGF level, (**b**) TrkA level, (**c**) p75NTR level, (**d**) pErk1/2/Erk1/2 ratio, in frontal cortex of control adolescent (C), fructose-fed adolescent (F), young-adult control rescued (CR), young-adult fructose-rescued (FR) rats. Data are the means ± SD of 8 rats/group. *P < 0.01, **P < 0.01, ***P < 0.001 versus C rats. ##*P* < 0,01, ###*P* < 0.001 versus CR rats. Source of variation: one-way Anova followed by Bonferroni post-test

According to previous works in human, rat, and mouse brain, reporting that mature NGF is absent while pro-NGF is the predominant species [53, 54], mature NGF was not detectable in any frontal cortex samples analyzed. Moreover, no significant difference between the experimental groups was found in the levels of its precursor pro-NGF (Fig. 3a). Interestingly, the protein level of TrkA, the receptor of NGF, was increased in F rats respect to C [*F* (3, 28) = 3.769; *P* < 0.05], while no difference was detected in FR compared to CR, after the sugar removal from the diet (Fig. 3b). Furthermore, no changes were observed in the amount of the low-affinity NGF receptor p75NTR (Fig. 3c). In addition, the phosphorylation degree of both Erk1 [*F* (3, 28) = 7.93; *P* < 0.01], and Erk2 [*F* (3, 28) = 14.16; *P* < 0.001], significantly increased in F compared to C rats (Fig. 3d), with persistent increased levels in FR compared to CR rats (*P* < 0.01 and *P* < 0.001, respectively).

**Fig. 4:**
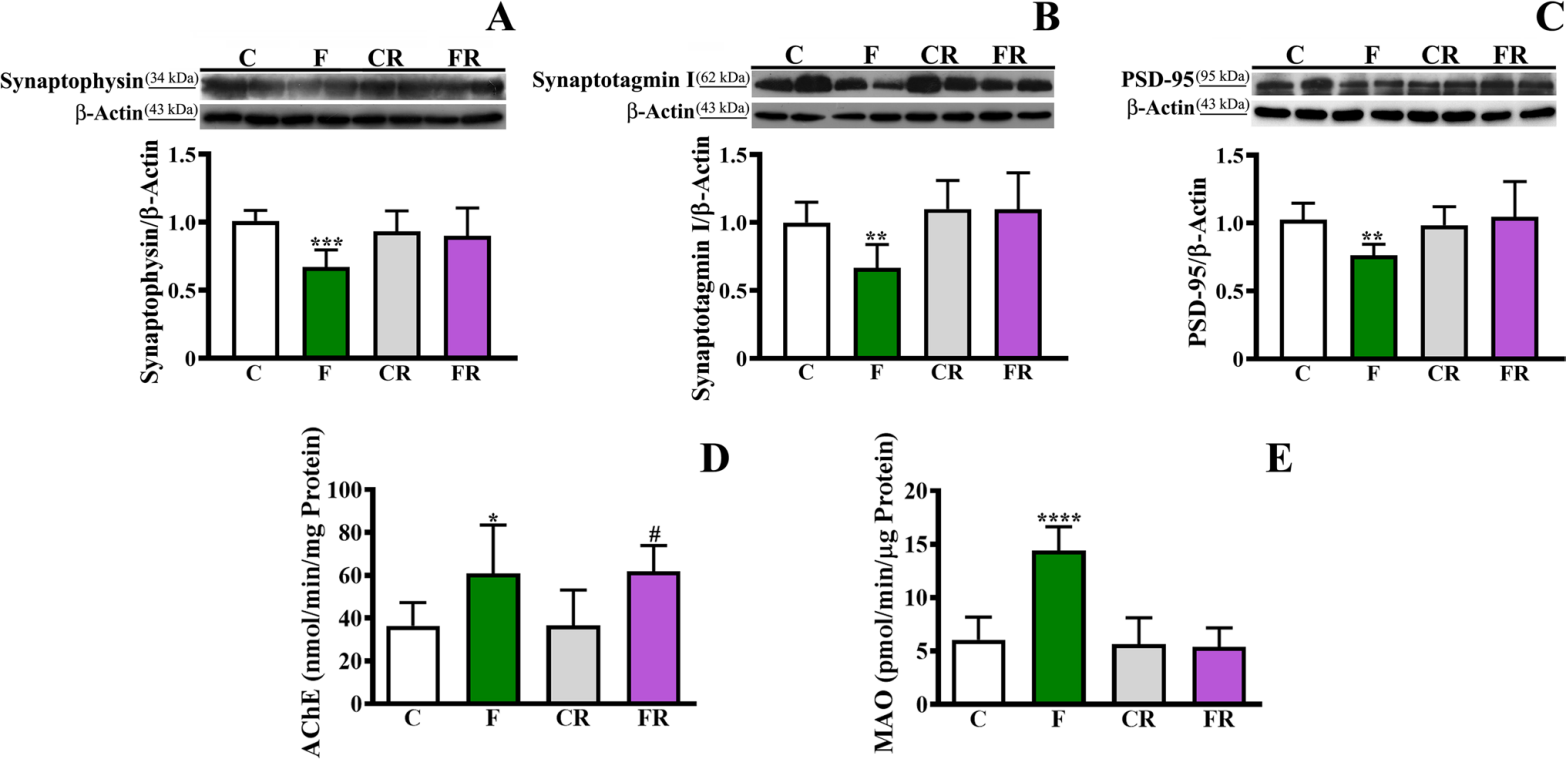
Evaluation of synaptic proteins, acetylcholinesterase and monoamino oxidase activity in frontal cortex. (**a**) Synapthophysin level (representative western blot and densitometric analysis), (**b**) synaptotagmin I level (representative western blot and densitometric analysis), (**c**) PSD-95 level (representative western blot and densitometric analysis), (**d**) AChE activity, and (**e**) MAO activity in frontal cortex of control adolescent (C), fructose-fed adolescent (F), young-adult control rescued (CR), young-adult fructose-rescued (FR) rats. Data are the means ± SD of 8 rats/group. *P < 0.05, **P < 0.01, ***P < 0.001 ****P < 0.0001 versus C rats. #*P* < 0.05 versus CR rats. Source of variation: one-way Anova followed by Bonferroni post-test

We further investigated the impact of fructose on the amounts of two pre-synaptic proteins, namely synaptophysin and synaptotagmin I, and the post-synaptic protein PSD-95, which play a key role in synaptic plasticity [55]. The fructose-rich diet led to decreased levels of all the three proteins [synaptophysin, *F* (3, 28) = 6.601; *P* < 0.001; synaptotagmin I, *F* (3, 28) = 6.978; *P* < 0.01; PSD-95, *F* (3, 28) = 5.452; *P* < 0.01], while the switch to control diet rescued their amount (Fig. 4a–c).

The effect of the short-term fructose diet on the activity of AChE, a pivotal enzyme involved in the regulation of cholinergic pathway [56], was also evaluated. As shown in Fig. 4d, fructose feeding resulted in a significant increase of AChE activity of F compared to C rats [*F* (3, 28) = 4.36; *P* < 0.05]. This increase was also found in FR rats compared to CR (*P* < 0.05). Notably, the activity of MAO, a central player in the modulation of monoamine neurotransmitters level [57], increased in F rats compared to C rats [*F* (3, 28) = 29.12; *P* < 0.0001; Fig. 4e), with no significant change between FR and CR rats.

### Metabolites and Neurotransmitters

**Fig. 5:**
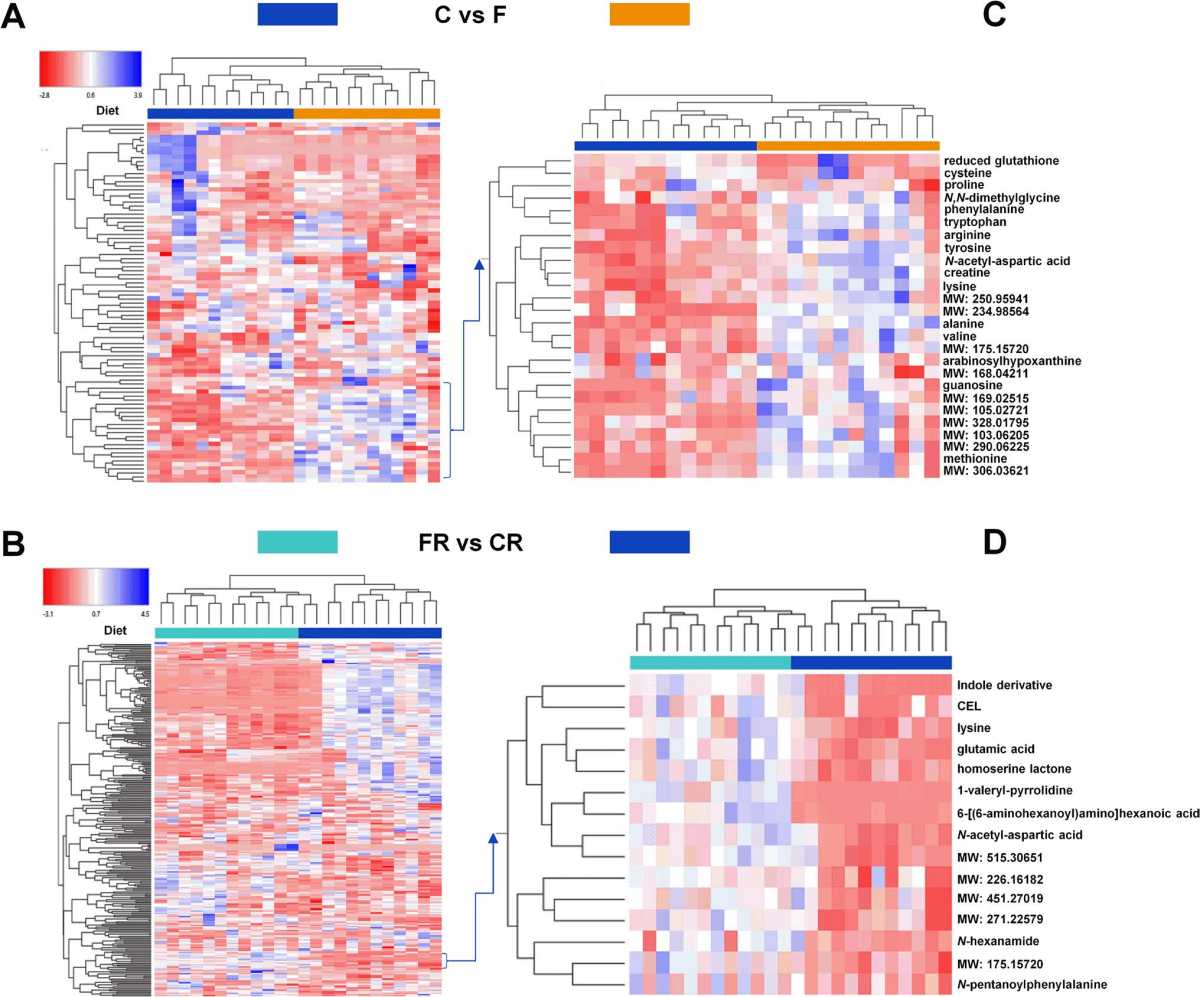
Metabolomic analysis of frontal cortex. (**a**) Heat-map hierarchical cluster analysis of metabolites detected in frontal cortex of fructose-fed adolescent (F, orange) and control adolescent (C, blue) rats. (**b**) Heat-map hierarchical cluster analysis of metabolites detected in frontal cortex of young-adult fructose-rescued (FR, light blue) and young-adult control rescued (CR, blue) rats. Dendrograms on the left report the molecular grouping and distance between molecular classes, while dendrograms on the top report the sample grouping. Corresponding iterative zoomed regions on the right (panels **c** and **d**) report identified molecules according to the number of matched over-represented compounds as result of differential analysis processing node within Compound Discoverer software (Supplementary Figure 2, and Supplementary Tables 2 and 3). Analytes were identified with a metabolite identification level of 2 as in the case that compound name was used and 4 in the case where only the calculated molecular weight (MW) was reported, as defined by Metabolomics Standards Initiative (MSI) [58]. Normalized area ranges and Eculidean distance were used in all cases; scaled expression values of each range are plotted in red to blue through white color scale

Polar hydrophilic metabolites were isolated from frontal cortex homogenates, purified by using protein and phospholipids cartridges, and finally measured by using HILIC coupled to high-resolution tandem mass spectrometry according to the nature and duration of the intervention study. To point out the role of fructose intake on small molecules fingerprinting, the C rat group was compared with the F one, while CR was matched to FR. Results are reported in the heat-maps (Fig. 5). Figure 5a highlighted a clear discrimination between fructose-diet (orange line) and control diet (blue line) through Euclidean distance between over- and down-represented analyte intensities in the centred and scaled area range − 2.8 and 3.9, as related to the *m/z* current ion associated with 89 molecules. Supplementary Table 2 shows over-represented compounds reported in Fig. 5c as well as the corresponding analytical performance and fold change values, along with their respective MSI (metabolomic standard initiative) levels [58]. Sample clustering was outlined in an explorative PCA combined with loading plots and differential analysis, which highlighted (in light blue) the compounds (Supplementary Table 2) having a log2 fold change higher than 0.5 and a negative log10 of the *p*-value higher than 0.05 (Supplementary Figure 2a).

In a similar way, polar hydrophilic metabolites differentiated rescue fructose-diet (light blue arrow, FR) and rescue control diet (blue arrow, CR) (Fig. 5b) when considering up to 272 over- and down-represented compounds. Supplementary Table 3 shows over-represented compounds in FR rats reported in Fig. 5d with a log2 fold change higher than 0.5 and with a negative log10 of the *p*-value higher than 0.05, as a result of the differential analysis (Supplementary Figure 2b). PCA illustrates how loadings plot contributed to the spatial arrangement of FR and CR samples, confirming results obtained through heat-map and hierarchical clustering (Supplementary Figure 2b).

Then, each of the over-represented compounds differentiated by the kind of diet (Supplementary Tables 2 and 3) was included in Metabolika node within Compound Discoverer with the aim to identify the most influenced metabolic pathways. Results obtained after loading the increased abundance of metabolites in Fig. 5c pinpointed metabolic pathways involving aromatic amino acids, namely tyrosine, phenylalanine, and tryptophan, along with sulphur compounds (cysteine and reduced glutathione) and other derivatives.

On the other hand, loading of data from the second heat-map deriving from differential analysis on rescue diet (Fig. 5d) outlined metabolic pathways associated with some polar basic amino acids, such as lysine and CEL, secondary amides and other acid compounds, like aspartic acid derivatives and glutamic acid (Fig. 5d). In this view, we hypothesized that the metabolism of aromatic amino acids, lysine, and glutamic acid can be the focus of high fructose diet. This hypothesis is in accordance with a previous observation [59], which reported that amino acids linked to tricarboxylic acid cycle, such glutamate and aspartate, undergo specific enrichment when young rats are fed with a high-fructose diet. Furthermore, a coherent alteration of the lysine catabolism was recently observed in diurnal metabolic pathways impacted by high-fat diet, suggesting that fructose intake can similarly exacerbate the alteration of amino acid metabolism [60].

**Fig. 6:**
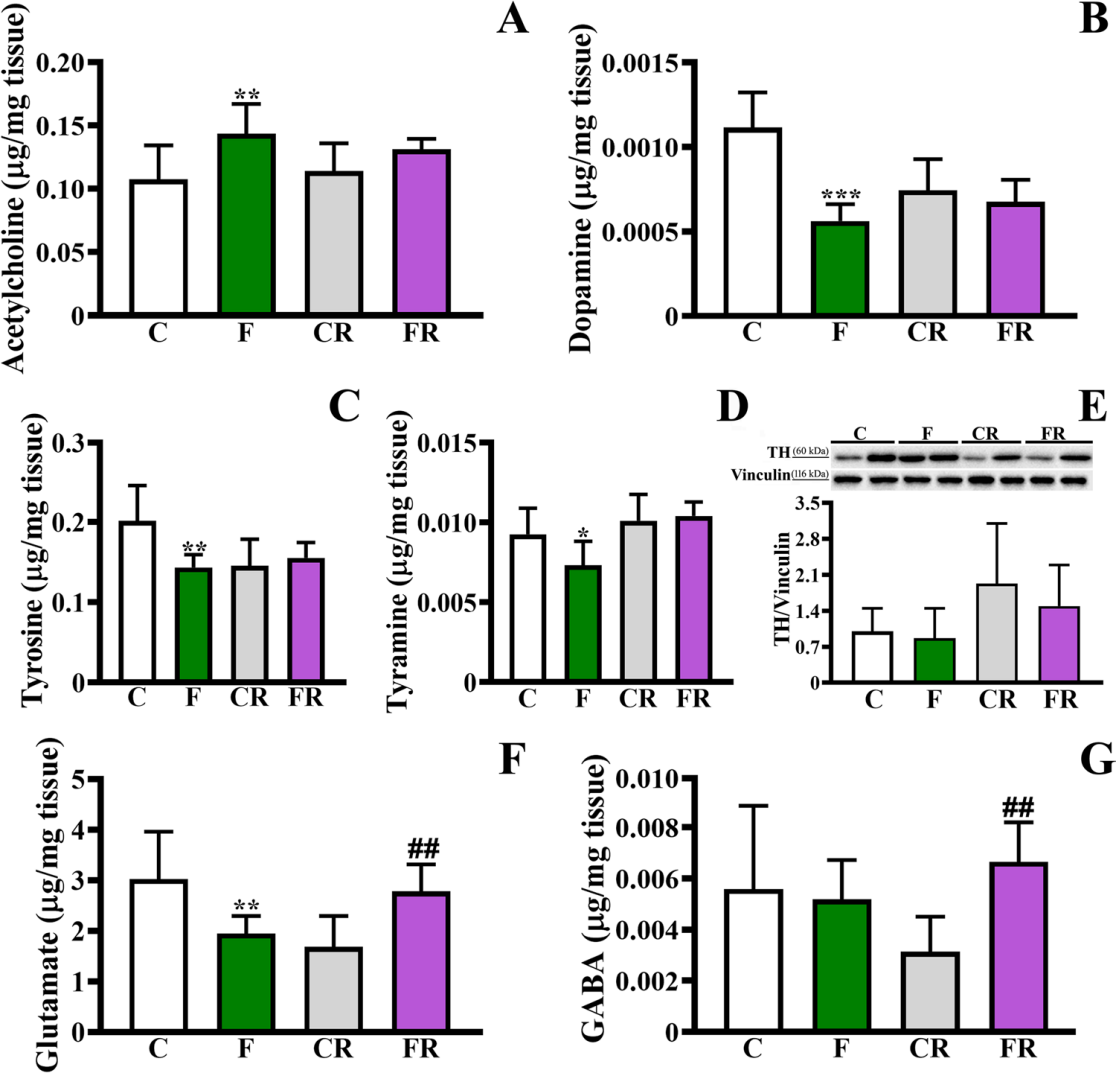
Evaluation of neurotransmitters and tyroxine hydrolase in frontal cortex. (**a**) Acetylcholine amount, (**b**) dopamine amount, (**c**) tyrosine amount, (**d**) tyramine amount, (**e**) tyroxine hydroxylase level (representative western blot and densitometric analysis), (**f**) glutamate amount, (**g**) GABA amount in frontal cortex of control adolescent (C), fructose-fed adolescent (F), young-adult control rescued (CR), young-adult fructose-rescued (FR) rats. Data are the means ± SD of 6 rats/group. *P < 0.01, **P < 0.01, ***P < 0.001 versus C rats. ##*P* < 0,01 versus CR. Source of variation: one-way Anova followed by Bonferroni post-test

Based on the above-reported untargeted analysis (Fig. 5), we then focused on the targeted quantification of six metabolites, based on full scan high resolution acquisition and the use of authentic reference standards, to highlight quantitative differences between C and F rats, and between CR and FR. Indeed, a major aim of the study was to evaluate the quantitative impact of the fructose diet on the profile of the major neurotransmitters (Fig. 6). We observed that the concentration of acetylcholine (ACh), one of the main neuromodulators of the central nervous system regulating individual attention, learning, and memory [61], was significantly higher in F rats compared to C [*F* (3, 20) = 4.202; *P* < 0.01], and this increase was rescued in FR (Fig. 6a). Conversely, the levels of dopamine, the neurotransmitter regulating the food eating reward circuit, motor activity, and emotion [62, 63], were reduced in F rats compared to C ones [*F* (3, 20) = 11.62; *P* < 0.001; Fig. 6b], and no difference between CR and FR groups was observed. Similarly, levels of both dopamine precursors tyrosine and tyramine decreased in F compared to C rats [tyrosine, *F* (3, 20) = 6.788, *P* < 0.01; tyramine, F (3, 20) = 6.509, *P* < 0.05; Fig. 6c–d], and returned to control values after the switch to the standard diet. On the other hand, no changes in the amounts of TH, the enzyme catalysing the rate-limiting step of catecholamine biosynthesis [64], were observed between F and C groups (Fig. 6e). Interestingly, the levels of the most common neurotransmitter in the CNS, namely glutamate [65], were lower in F group with respect to C one [*F* (3, 20) = 7.047; *P* < 0.01; Fig. 6f]. A different trend was observed after the switch to control diet, as FR showed higher glutamate levels compared to CR (Fig. 6f; *P* < 0.01). These results demonstrated that fructose intake is associated with a dysregulation of glutamate metabolism, which is further observed, but with an opposite quantitative trend, after switching to control diet. Finally, GABA levels did not differ between C and F groups, while increased amounts were observed in FR compared to CR [*F* (3, 20) = 3.477; *P* < 0.01; Fig. 6g].

## Discussion

Different lines of evidence have recently highlighted the fructose impact on brain metabolic alterations [13, 23], but it remains unclear whether the observed sugar-induced adverse effects are limited exclusively to the period of increased intake or are persistent even when it is eliminated from the dietary regimen. We recently reported that the fructose-rich diet impacts different metabolic parameters by inducing systemic inflammation, hepatic insulin resistance, increase of plasma triglycerides [66], and “leaky gut” [67], which persisted after switching to control diet. Sugar-driven systemic inflammation and metabolic dysfunction could impact on brain as well. Here we studied, by using the same experimental design, the metabolic effects of the sugar in frontal cortex of adolescent animal model, since, to our knowledge, poor information is available on this issue.

The fructose diet was associated with increased levels of the fructose transporter Glut-5 in rat frontal cortex, and a concomitant increase of fructose and uric acid, which were suggestive of an enhanced fructose metabolism therein. The change of Glut-5 and metabolite levels were paralleled by an inflammatory status. A similar pattern was previously found in the hippocampus [47], thus showing that dietary fructose reaches the brain and is utilized in several cerebral areas. Augmented levels of uric acid were also reported to elicit oxidative stress [68–70] and consistently we found a higher extent of oxidative damage to proteins. Inflammation and oxidative stress are often related to a mitochondrial dysfunction [71, 72], and indeed we evidenced a corresponding general impairment of the mitochondrial oxidative capacity not linked to a lower organelle mass, since PGC-1α level was not altered. All above-mentioned fructose-dependent effects on mitochondrial activity were reverted when this sugar was removed from the dietary regimen.

More importantly, higher advanced glycation end-products (CML and CEL) were found in fructose-fed rats, in line with previous works showing that fructose can lead to the formation of highly reactive intermediate products as α-dicarbonyls [73], which in turn can favor the formation of advanced glycation end-products, as CML and CEL [74], thus acting as potent glycating agent [75] and negatively impacting the brain function [13, 76, 77]. Interestingly, the observed augmented levels of CML and CEL in fructose-fed rats were not restored to control values in FR group, suggesting the persistence of the sugar-associated effects.

Given the key role of neurotrophins in brain activity [50], we focused on the analysis of BDNF and NGF and we observed that the level of BDNF and its receptor TrkB was lower in fructose-fed rats. This result is in line with previous findings obtained in adult rats experiencing a long- or short-term fructose intake [25, 27], highlighting this neurotrophin as a crucial target of fructose diet independently from the age. The early reduction of BDNF in adolescent rats and its persistent decrease later was corroborated by the observation of lower activation of its downstream effector, namely CREB, both in F compared to C rats and in FR compared to CR counterparts. Since CREB is implicated in the transcription of genes essential for synaptic plasticity [78], the persistent reduction in its phosphorylated form is suggestive of a possible long-term brain impairment. The sugar-induced impairment of BDNF signaling might be compensated, at least in part, by the increase of TrkA and activation of its downstream transducers Erk1/2. This process is regulated by pro-NGF and critically depends on the balance of TrkA and p75NTR [79, 80]. This pathway is indeed committed to prevent apoptotic signals, with a positive effect on neuronal survival and neurite outgrowth [54].

The persistent impairment of BDNF-CREB signaling prompted us to investigate the issue of synaptic functioning. A decrease of pre- (synaptophysin, and synaptotagmin I) and post-(PSD-95) synaptic proteins following fructose dietary treatment was found, and this change was rescued after the switch to the standard diet, suggesting that the homeostasis of these proteins is restored more rapidly than that of BDNF. To gain further insight into synaptic physiology, we also measured the activity of AChE and MAO, as well as the levels of important neurotransmitters involved in synaptic transmission. In particular, increased activity of AChE and MAO was detected in fructose-fed rats. While increased MAO rescued after switching to standard diet, AChE increase persisted after fructose removal from the diet. In this context, it is worth mentioning that the enhanced AChE activity in the hippocampus and prefrontal cortex was previously proposed as an early event linked to hypercholesterolemia- or high-fat diet-induced alterations in cognitive function [81–83]. Notably, despite the enhanced AChE activity, we detected increased levels of ACh in fructose-fed rats. In this regard, previous studies showed that an abnormal intake of fructose provokes an immediate drop in the ATP/AMP ratio, a decreased acetyl-CoA carboxylase activity, and a consequent lowering of malonyl-CoA levels [84]. Accordingly, it can be hypothesized that the increased ACh levels after fructose intake might derive from the increased availability of acetyl CoA, consequent to the decreased activity of acetyl-CoA carboxylase. In this sense, the higher AChE activity may represent an adaptive response to prevent prolonged ACh signaling. The increase of ACh levels in F rats suggests that a short-term fructose enriched diet may disrupt cholinergic signaling and predispose adolescents to altered states, such as anxiety disorders and depression, also corroborated by BDNF and dopamine reduction. The observed decrease of dopamine, which regulates different brain functions such as reinforcement processing, motivation, and attention [85], might be ascribed to both the corresponding enhanced MAO activity and the reduced amounts of tyrosine and tyramine. In agreement with our findings, previous studies have reported that HFCS can impair dopamine function in the absence of weight gain or increased fat consumption [10]. As a reduced dopamine activity has been implicated in reduced energy expenditure [86, 87], changes in dopamine metabolism were proposed to precede and possibly contribute to obesity in the long-term period [10].

A general alteration of some metabolic pathways related to specific aromatic or sulfur-containing amino acids, lysine and glutamic acid was also deduced from F vs C, and FR vs CR comparisons. Glutamate is the principal excitatory neurotransmitter involved in learning, memory and cognition, and any unbalance of its turnover may have severe consequences [65]. Decreased glutamate levels in fructose-fed rats and then an increase of this neurotransmitter in FR rats compared to CR counterpart might be suggestive of a glutamatergic persistent dysregulation after fructose intake, which might prelude to long term dysfunction. Indeed, glutamatergic dysregulation has already been reported being an important contributor to different neurological pathologies [65].

ACh, dopamine, and glutamate changes observed in fructose-fed rats were not paralleled by GABA alterations. Since higher levels of glutamate were observed in FR rats, we cannot exclude that the concomitant increase of GABA amount may represent a compensatory mechanism to prevent possible dysfunction related to concomitant quantitative changes of its precursor.

**Fig. 7:**
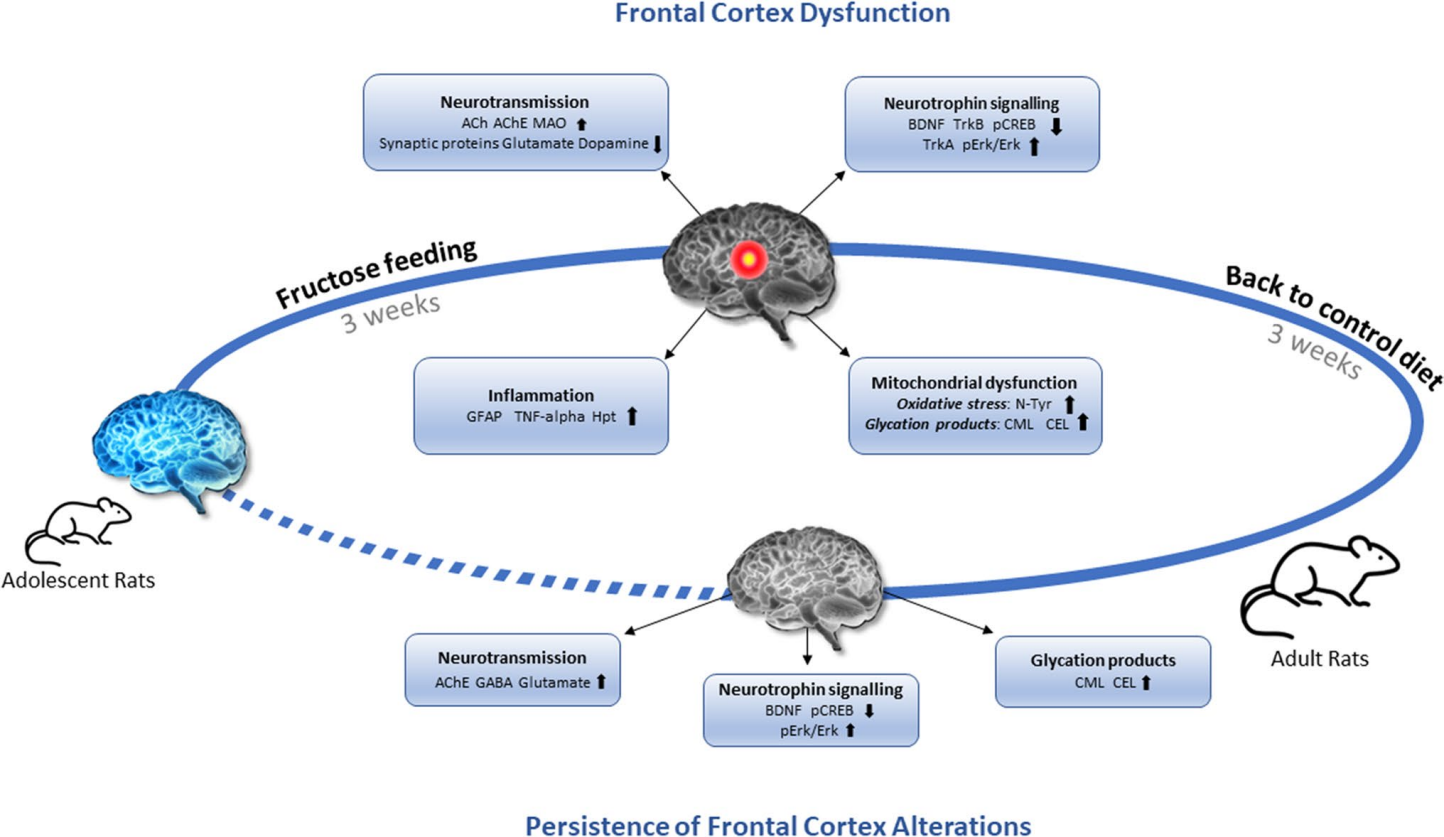
Fructose impact on brain health of adolescent rats and persistence of its effect after switching to a control diet. Up arrows indicate parameters increased and down arrows indicate parameters reduced. ACh, acetylcholine; AChE, acetylcholinesterase; MAO, monoamine oxidase; BDNF, brain derived neurotrophic factor; TrkB, tropomyosin receptor kinase B; TrkA, tropomyosin receptor kinase A; pErk1/2, pospho-extracellular signal-regulated kinase 1/2; Erk1/2, extracellular signal-regulated kinase 1/2; pCREB, pospho-cAMP-response element binding protein; GFAP, glial fibrillary acidic protein; Hpt, haptoglobin; CML, *N*ε-carboxymethyllysine; CEL, *N*ε-carboxyethyllysine

In conclusion, this study demonstrates that the fructose feeding causes a perturbation of various biochemical machineries involved in brain metabolism and function, such as neurotrophins signaling and a consequent possible modification of excitatory/inhibitory neurotransmitter balance, which is essential for the proper functioning of the central nervous system (Fig. 7). Undoubtedly, these data also point out that adolescence represents a developmental window of vulnerability, in which extreme attention should be devoted to limit an excessive consumption of industrial and processed sweet food, since it can impact brain physiology not only immediately but also in the long term. Since previous reports showed different susceptibilities of males and females to fructose supplementation [88, 89], future experiments will be critical to clarify whether the sugar-adverse effects and/or their persistence in brain can be different depending on the sex.

## Supplementary Information

Below is the link to the electronic supplementary material.

Supplementary file1 (DOCX 805 KB)

## Data Availability

All data supporting the findings of this study are available within the article and its supplementary information files.
