# Supplementary material for: Long-Lasting Impact of Sugar Intake on Neurotrophins and Neurotransmitters from Adolescence to Young Adulthood in Rat Frontal Cortex
Source: Mol Neurobiol. 2022 Nov 17;60(2):1004–20. doi: 10.1007/s12035-022-03115-8 (PMC9849314; doi:10.1007/s12035-022-03115-8)
Supplement: Supplementary file 1 — Supplementary file1 (DOCX 805 KB) [file 12035_2022_3115_MOESM1_ESM.docx]

**Long-lasting impact of sugar intake on neurotrophins and neurotransmitters from childhood to young adulthood in rat frontal cortex**

Maria Stefania Spagnuolo^1^, Arianna Mazzoli^2^, Martina Nazzaro^2^, Antonio Dario Troise^1^, Cristina Gatto^2^, Claudia Tonini^3^, Mayra Colardo^4^, Marco Segatto^4^, Andrea Scaloni^1^, Valentina Pallottini^3,5^, Susanna Iossa^2^, Luisa Cigliano^2*^

^1^Institute for the Animal Production System, National Research Council, Portici, Italy.

^2^Department of Biology, University of Naples Federico II, Naples, Italy.

^3^Department of Science, Biomedical and Technology Science Section, University Roma Tre, Rome, Italy.

^4^Department of Biosciences and Territory, University of Molise, Pesche, Italy.

^5^Neuroendocrinology Metabolism and Neuropharmacology Unit, IRCSS Fondazione Santa Lucia, Rome, Italy.

*Correspondence: Prof. Luisa Cigliano, Department of Biology, University of Naples Federico II, Complesso Universitario Monte S. Angelo, Edificio 7, Via Cintia - I-80126 Napoli ITALY. E-mail: luisa.cigliano@unina.it, phone number:+39-081-2535244

**Supplementary Table 1.** Primary and secondary antibodies dilutions used for Western blotting analysis

| Antigen | Primary Antibody | Secondary Antibody |
| --- | --- | --- |
| Glut-5 | Invitrogen (PA5-80023); 1:1,000**^a^** | GAR-HRP IgG; 1:45,000**^b^** |
| Glut-4 | Santa Cruz Biotechnology (sc-53566); 1:1,000**^a^** | GAM-HRP IgG; 1:15,000**^b^** |
| GFAP | Cell Signalling Technology (#12389); 1:1,000**^c^** | GAR-HRP IgG; :150,000**^d^** |
| Synaptophysin | AB9272; Merk-Millipore; 1:150,000**^a^** | GAR-HRP IgG; 1:40,000**^e^** |
| Synaptotagmin I | Cell Signalling Technology (#14588); 1:1,000**^c^** | GAR-HRP IgG; :200,000**^f^** |
| PSD-95 | Cell Signalling Technology (#2507); 1:1,000**^c^** | GAR-HRP IgG; 1:70,000**^f^** |
| BDNF | Santa Cruz Biotechnology (sc-546); 1:500**^c^** | GAR-HRP IgG; 1:70,000**^d^** |
| pro-BDNF | Santa Cruz Biotechnology (sc-546); 1:1,000**^c^** | GAR-HRP IgG; 1:10,000**^d^** |
| PGC-1α | Merk-Millipore (#AB3242);  1:2,000**^a^** | GAR-HRP IgG; 1:20,000**^b^** |
| TrkB | Santa Cruz Biotechnology (sc-3772218); 1:2,000**^a^** | GAR-HRP IgG; :140,000**^b^** |
| pErk1/2 | Cell Signalling Technology (#9101S); 1:1,000**^a^** | GAR-HRP IgG; :150,000**^b^** |
| Erk1/2 | Cell Signalling Technology (#9102); 1:1,000**^a^** | GAR-HRP IgG; :170,000**^b^** |
| TH | Santa Cruz Biotechnology (sc-25269); 1:1,000**^d^** | GAM-HRP IgG; 1:10,000**^d^** |
| TrkA | Santa Cruz Biotechnology (sc-118); 1:1,000**^d^** | GAR-HRP IgG; 1:10,000 **^d^** |
| p75NTR | Santa Cruz Biotechnology (sc-271708); 1:1,000**^d^** | GAM-HRP IgG; 1:10,000**^d^** |
| NGF | Santa Cruz Biotechnology (sc-365944); 1:1,000**^d^** | GAM-HRP IgG; 1:10,000**^d^** |
| β -Actin | Sigma-Aldrich (A2228);  1:1,000**^c^** | GAM-HRP IgG; 1:35,000**^e^** |
| Vinculin | Sigma-Aldrich (V9131);  1:10,000**^d^** | GAM-HRP IgG; 1:10,000**^d^** |

**GAR-HRP**: Goat anti-rabbit Horseradish peroxidase-conjugated IgG (Immunoreagents, Raleigh, NC, USA; catalogue number GtxRb-003-DHRPX); **GAM-HRP**: Goat anti-mouse Horseradish peroxidase-conjugated IgG (Immunoreagents, Raleigh, NC, USA; catalogue number GtxMu-003-DHRPX); **T-TBS**: 130 mM NaCl, 20 mM Tris-HCl, 0.05% Tween, pH 7.4; **^a^** T-TBS containing 3% w/v BSA; **^b^:** T-TBS containing 2% w/v BSA; **^c^**: T-TBS containing 3% v/v non-fat milk; **^d^:** T-TBS containing 5% v/v non-fat milk; **^e^**: T-TBS containing 1% v/v non-fat milk; **^f^**: T-TBS containing 3% v/v non-fat milk.

**Supplementary Table 2. Differentially over-represented compounds as determined by HILIC-Fourier transform mass spectrometry (FTMS) untargeted analysis in control adolescent (C) and fructose-fed adolescent (F) rats.** Error (Δ ppm) was calculated as the ratio between the difference of the theoretical mass minus the experimental mass and the theoretical mass, multiplied per one million. RT, retention time; metabolite identification level was defined according to Metabolomics Standards Initiative (MSI) (Sumner et al., 2007); calc. MW, calculated molecular weight.

| Name | Formula | Δ [ppm] | Calc. MW | RT [min] | Ratio: (F) / (C) | Log2 Fold Change: (F) / (C) | P-value: (F) / (C) | MSI level |
| --- | --- | --- | --- | --- | --- | --- | --- | --- |
| Creatine | C4 H9 N3 O2 | -3.39 | 131.06903 | 7.209 | 1.788 | 0.84 | 1.55986E-06 | 2 |
| N,N-Dimethylglycine | C4 H9 N O2 | -4.74 | 103.06284 | 6.477 | 1.464 | 0.55 | 0.001806643 | 2 |
| N-Acetyl-L-aspartic acid | C6 H9 N O5 | -2.26 | 175.04768 | 7.409 | 1.95 | 0.96 | 1.1877E-05 | 2 |
| Arabinosylhypoxanthine | C10 H12 N4 O5 | -1.92 | 268.08026 | 6.635 | 1.487 | 0.57 | 0.000377137 | 2 |
| DL-Phenylalanine | C9 H11 N O2 | -1.91 | 165.07866 | 7.203 | 1.758 | 0.81 | 0.013029206 | 2 |
| L-(+)-Valine | C5 H11 N O2 | -3.32 | 117.07859 | 7.32 | 1.783 | 0.83 | 0.00138459 | 2 |
|  | C2 H8 N3 O4 P | -1.11 | 169.02505 | 7.188 | 1.553 | 0.64 | 0.000743623 | 4 |
| L-(-)-Methionine | C5 H11 N O2 S | -2.84 | 149.05063 | 7.334 | 2.024 | 1.02 | 0.009802015 | 2 |
| L-(+)-Alanine | C3 H7 N O2 | -4.46 | 89.04728 | 7.593 | 1.819 | 0.86 | 1.14045E-09 | 2 |
| L-Tyrosine | C9 H11 N O3 | -2.08 | 181.07352 | 7.348 | 1.731 | 0.79 | 1.60125E-05 | 2 |
|  | C10 H6 N6 O6 | 4.36 | 306.03621 | 6.622 | 1.568 | 0.65 | 0.000444015 | 4 |
| Guanosine | C10 H13 N5 O5 | -1.58 | 283.09122 | 7.453 | 2.404 | 1.27 | 1.14514E-05 | 2 |
| L-Proline | C5 H9 N O2 | -3.74 | 115.0629 | 7.954 | 32.442 | 5.02 | 0.000354262 | 2 |
|  | C7 H3 N5 P2 S | 3.87 | 250.95941 | 7.423 | 2.698 | 1.43 | 7.84582E-07 | 4 |
| DL-Arginine | C6 H14 N4 O2 | -1.82 | 174.11136 | 7.308 | 1.45 | 0.54 | 0.002900577 | 2 |
|  | C7 H10 N6 O7 | 3.97 | 290.06225 | 6.616 | 1.571 | 0.65 | 0.013255 | 4 |
| L-Lysine | C6 H14 N2 O2 | -2.75 | 146.10513 | 7.39 | 1.884 | 0.91 | 0.001095626 | 2 |
| L-Tryptophan | C11 H12 N2 O2 | -1.04 | 204.08967 | 7.197 | 1.922 | 0.94 | 0.000201327 | 2 |
|  | C8 H N3 O6 | -3.8 | 234.98564 | 7.419 | 2.115 | 1.08 | 3.0846E-05 | 4 |
|  | C11 H8 N2 O10 | 0.17 | 328.01795 | 6.633 | 1.462 | 0.55 | 0.010055157 | 4 |

**Supplementary Table 3. Differentially over-represented compounds as determined by HILIC-Fourier transform mass spectrometry (FTMS) untargeted analysis in young-adult control rescued (CR) and young-adult fructose-rescued (FR) rats.** Error (Δ ppm) was calculated as the ratio between the difference of the theoretical mass minus the experimental mass and the theoretical mass, multiplied per one million. RT, retention time; metabolite identification level was defined according to Metabolomics Standards Initiative (MSI) (Sumner et al., 2007); calc. MW, calculated molecular weight.

| Name | Formula | Δ [ppm] | Calc. MW | RT [min] | Ratio: (FR) / (CR) | Log2 Fold Change: (FR) / (CR) | P-value: (FR) / (CR) | MSI level |
| --- | --- | --- | --- | --- | --- | --- | --- | --- |
| L-Glutamic acid | C5 H9 N O4 | -3.07 | 147.05271 | 8.462 | 1.507 | 0.59 | 7.10273E-07 | 2 |
| N-Acetyl-L-aspartic acid | C6 H9 N O5 | -1.7 | 175.04777 | 7.387 | 1.945 | 0.96 | 2.69033E-05 | 2 |
| 1-valeryl-pyrrolidine, | C9 H17 N O | -3.94 | 155.1304 | 2.352 | 76.734 | 6.26 | 2.59576E-15 | 2 |
| n-Hexanamide | C6 H13 N O | -2.68 | 115.09941 | 2.268 | 1.521 | 0.61 | 0.007465124 | 2 |
|  | C13 H35 N6 P3 S2 | 3.1 | 432.15908 | 8.463 | 1.531 | 0.61 | 7.8472E-06 | 4 |
| 6-[(6-Aminohexanoyl)amino]hexanoic acid | C12 H24 N2 O3 | -0.86 | 244.17848 | 3.098 | 19.622 | 4.29 | 3.21218E-07 | 2 |
| L-Lysine | C6 H14 N2 O2 | -2.04 | 146.10523 | 7.401 | 2.332 | 1.22 | 2.29768E-05 | 2 |
| Indole derivative | C16 H23 N5 O | -4.67 | 301.18885 | 2.701 | 56.171 | 5.81 | 3.13247E-05 | 2 |
| CEL | C9 H18 N2 O4 | -0.17 | 218.12662 | 7.013 | 2.359 | 1.24 | 0.001400264 | 2 |
|  | C8 H15 N2 O5 P3 S | 0.79 | 343.99172 | 6.665 | 1.532 | 0.62 | 0.000199173 | 4 |
| L-homoserine lactone | C4 H7 N O2 | -3.36 | 101.04734 | 8.465 | 1.498 | 0.58 | 3.76852E-06 | 2 |
|  | C16 H39 N9 O2 P2 | -0.01 | 451.27019 | 3.813 | 1.508 | 0.59 | 0.01782836 | 4 |
|  | C14 H29 N3 O2 | -0.7 | 271.22579 | 4.475 | 1.563 | 0.64 | 0.018627875 | 4 |
|  | C9 H21 N O2 | -0.19 | 175.1572 | 3.363 | 1.987 | 0.99 | 0.000869172 | 4 |
| N-pentanoylphenylalanine | C14 H19 N O3 | -0.41 | 249.13639 | 3.334 | 2.207 | 1.14 | 9.66955E-06 | 2 |
|  | C16 H42 N5 P3 | 4.61 | 397.26714 | 2.165 | 1.441 | 0.53 | 0.011179231 | 4 |
|  | C18 H47 N9 O2 P2 S | 3.18 | 515.30651 | 2.248 | 2.544 | 1.35 | 0.000628253 | 4 |
|  | C12 H22 N2 O2 | -0.05 | 226.16812 | 3.424 | 2.933 | 1.55 | 0.000433627 | 4 |

**Supplementary Table 4. Targeted zwitterionic HILIC-Fourier transform mass spectrometry (FTMS) analysis.** Error (Δ ppm) was calculated as the ratio between the difference of the theoretical mass minus the experimental mass and the theoretical mass, multiplied per one million. RT, retention time; GABA, γ-aminobutyric acid. Metabolite identification level, as defined according to Metabolomics Standards Initiative (MSI) (Sumner et al., 2007), was 1 for all the listed compounds upon matching with authentic reference analytical standards.

| **Compound Name** | **RT** | **Elemental composition** | ***m/z theoretical*** | ***m/z experimental*** | Δ ppm |
| --- | --- | --- | --- | --- | --- |
| Nε-carboxymethyllysine | 7.2 | C_8_H_16_N_2_O_4_ | 205.11832 | 205.11801 | -1.5 |
| Nε-carboxyethyllysine | 7.5 | C_9_H_18_N_2_O_4_ | 219.13393 | 219.13407 | 0.6 |
| Glutamate | 8.5 | C_5_H_9_NO_4_ | 148.06043 | 148.06085 | 2.8 |
| GABA | 5.7 | C_4_H_9_NO_2_ | 104.07060 | 104.07051 | -0.9 |
| Tyrosine | 7.4 | C_9_H_11_NO_3_ | 182.08117 | 182.08132 | 0.8 |
| Tyramine | 6.7 | C_8_H_11_NO | 138.09134 | 138.09117 | -1.2 |
| Dopamine | 3.5 | C_8_H_11_NO_2_ | 154.08625 | 154.08658 | 2.1 |
| Acetylcholine | 4.9 | C_7_H_16_NO_2_^+^ | 146.11755 | 146.11781 | 1.8 |

**Supplementary Figure 1. Evaluation of Glucose and Glut-4 in rat frontal cortex**

**
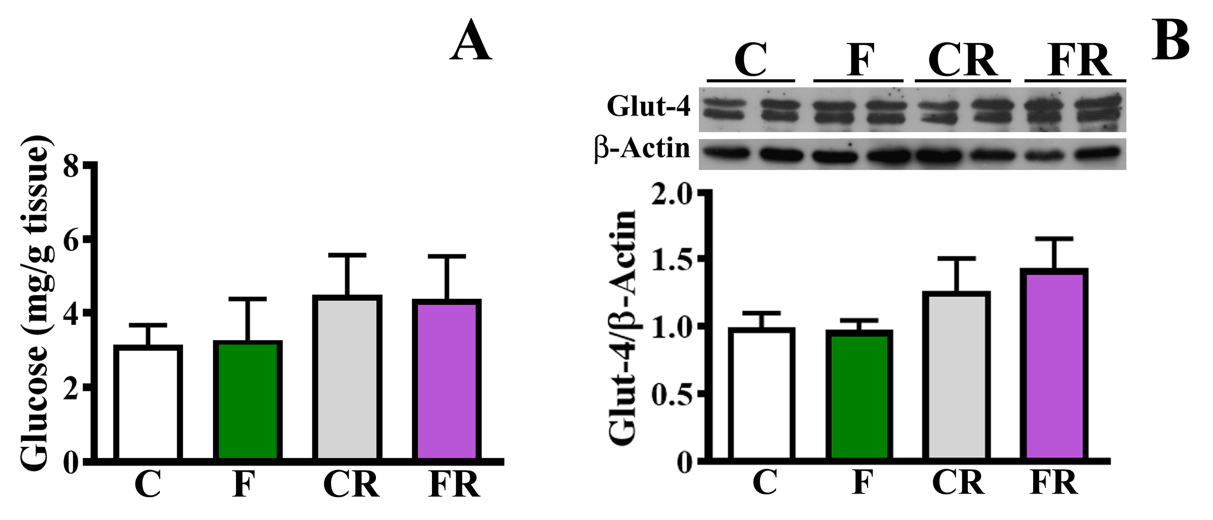
**

1. Glucose amount; b) Glut-4 level (representative western blot and densitometric analysis) in frontal cortex of control adolescent (C), fructose-fed adolescent (F), young-adult control rescued (CR), young-adult fructose-rescued (FR) rats. Data are the means ± SD of 8 rats/group.

**Supplementary Figure 2. Principal component analysis (PCA), loadings plot and volcano plot.** Panel A reports explorative PCA with control adolescent (C) and fructose-fed adolescent (F) rats, along with loadings plot and volcano plot based on differntial analysis with a p-value 0.05, while for log2 fold change 0.5 was used. All the metabolites reported in Supplementary Table 2 are shown in light blue. Panel B reports the PCA, loadings and volcano plots from young-adult control rescued (CR) and young-adult fructose-rescued (FR) rats. Light blue spotted points depict differentially over-represented metabolites listed in Supplementary Table 3. Due to the low number of metabolites down-represented in C vs F volcano plot (panel A), we only focused on over-represented metabolites.


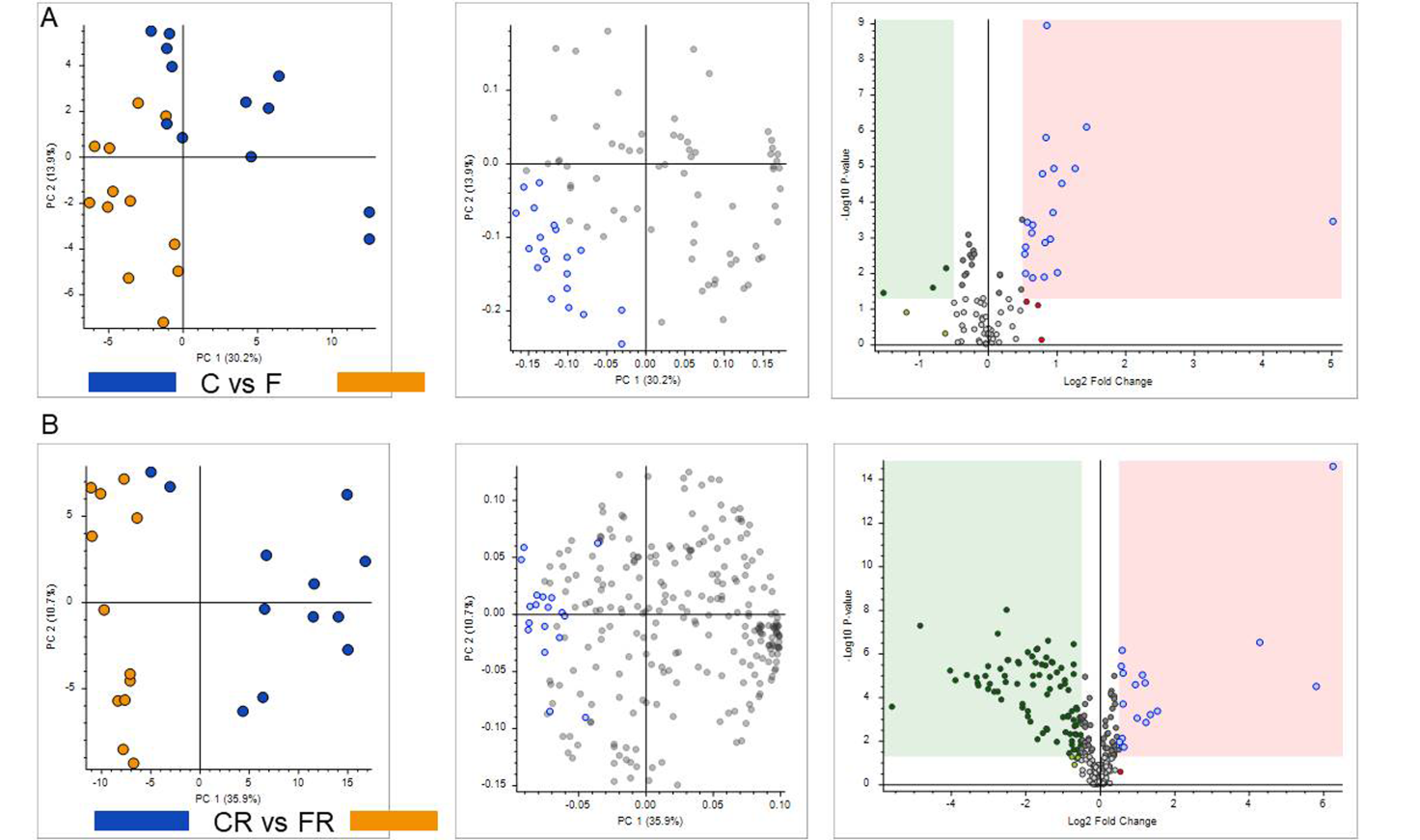


**Reference**

1. Sumner LW, Amberg A, Barrett D, Beale MH, Beger R, Daykin CA, Fan TW, Fiehn O, Goodacre R, Griffin JL, Hankemeier T, Hardy N, Harnly J, Higashi R, Kopka J, Lane AN, Lindon JC, Marriott P, Nicholls AW, Reily MD, Thaden JJ, Viant MR (2007) Proposed minimum reporting standards for chemical analysis Chemical Analysis Working Group (CAWG) Metabolomics Standards Initiative (MSI). Metabolomics 3(3):211-221. https://doi.org/10.1007/s11306-007-0082-2.
